# Comparison of Intravitreal Ranibizumab and Laser Photocoagulation in the Treatment of Type I Retinopathy of Prematurity in Malaysia: A One-Year Follow-Up Study

**DOI:** 10.7759/cureus.63712

**Published:** 2024-07-02

**Authors:** Jami Wardati H, Mustafa Khadijah, Mustafa Nurul-Farhana, Wahit Karimmah, Yoon Kit Ivan Lai, Md Razali Syahmi, Fiona Lee Min Chew, Jamalia Rahmat, Norhafizah Hamzah, Ismail Shatriah

**Affiliations:** 1 Department of Ophthalmology and Visual Science, School of Medical Sciences, Universiti Sains Malaysia, Kubang Kerian, MYS; 2 Ophthalmology Clinic, Hospital Universiti Sains Malaysia, Kota Bharu, MYS; 3 Department of Ophthalmology, Hospital Selayang, Batu Caves, MYS; 4 Department of Ophthalmology, Faculty of Medicine, Universiti Teknologi MARA, Puncak Alam, MYS; 5 Department of Ophthalmology, Universiti Malaya, Kuala Lumpur, MYS; 6 Department of Ophthalmology, Hospital Kuala Lumpur, Kuala Lumpur, MYS; 7 Department of Ophthalmology, Sunway Medical Centre Velocity, Kuala Lumpur, MYS; 8 Department of Ophthalmology, Hospital Tunku Azizah, Kuala Lumpur, MYS

**Keywords:** type i rop, retinopathy of prematurity, ranibizumab, anti-vegf, laser photocoagulation

## Abstract

Purpose: This study aimed to evaluate the treatment efficacy, anatomical outcomes, and refractive outcomes of laser photocoagulation (LPC) and intravitreal ranibizumab (IVR) in the treatment of type I retinopathy of prematurity (ROP) at one-year follow-up.

Methods: This is a retrospective study on the treatment of type I ROP and aggressive ROP (A-ROP) using LPC or IVR in three Malaysian hospitals providing pediatric ophthalmology services from January 2019 to December 2021. Information on gestational age, birth weight, ROP zone and stage, and underlying comorbidities was collected. Parameters for evaluating treatment efficacy include the time taken to achieve complete regression, the regression rate, and the reactivation rate. The anatomical and refractive outcomes were evaluated at one year of adjusted age.

Results: This study included 92 eyes from 46 infants. Of these, 42 eyes received LPC as the initial treatment, while 50 eyes underwent IVR. A higher percentage of infants with cardiovascular disease were treated with IVR (66.7%) compared to LPC (40%) (p<0.05). However, there were no significant differences in gestational age, birth weight, respiratory distress syndrome, sepsis, or intraventricular hemorrhage between the two treatment groups (p>0.05). Infants treated with LPC had a higher regression rate than those treated with IVR, but they were also significantly more myopic and had worse best-corrected visual acuity (BCVA). Conversely, infants treated with IVR experienced a significantly higher reactivation rate compared to those treated with LPC. Logistic regression analysis showed no significant associations between gestational age, birth weight, plus disease, zone 1 ROP, and the choice of initial treatment with the reactivation of ROP.

Conclusions: Both LPC and IVR effectively treat type I ROP in infants, with IVR yielding superior anatomical and refractive outcomes and LPC offering a lower reactivation rate. Understanding individual patient characteristics is crucial for treatment selection.

## Introduction

Retinopathy of prematurity (ROP) is a condition that affects premature infants due to disruption of the natural process of retinal vascularization, typically occurring in the last trimester of pregnancy. Identified as the primary cause of childhood blindness, the pathogenesis of ROP is linked to altered levels of vascular endothelial growth factor (VEGF), insulin-like growth factor I, oxygen tension, and various other contributing factors. These alterations lead to aberrant angiogenesis, resulting in retinal vascular pathology and subsequent irreversible retinal injury [1,2].

Significant advancements have transformed the screening, treatment, and understanding of ROP over the last four decades. Landmark studies such as Cryotherapy for ROP (CRYO-ROP) in 1988 and Early Treatment for ROP (ETROP) in 2004 have played pivotal roles in shaping treatment protocols for threshold and pre-threshold type I ROP. The CRYO-ROP trial demonstrated a significant reduction in total retinal detachment from 38.6% to 22.1% through cryotherapy [3]. Similarly, the ETROP study showcased the efficacy of prompt laser photocoagulation (LPC), reducing unfavorable anatomical outcomes from 15.6% to 9.1% [4]. Further advancements, such as those seen in the Bevacizumab Eliminates the Angiogenic Threat of ROP (BEAT-ROP) and Ranibizumab versus LPC for the treatment of very low birth weight infants with ROP (RAINBOW) trials, have demonstrated a reduced incidence of high myopia after anti-VEGF therapy compared to LPC in a two-year follow-up period [5,6].

In the past decade, anti-VEGF therapy has emerged as an effective treatment option for ROP [5-8]. Ranibizumab, a fully humanized monoclonal antibody fragment with a molecular weight of 48 kDa and a serum half-life of approximately 5.8 days, has played a significant role in this emergence [9]. Following its introduction, intravitreal ranibizumab (IVR) has become the preferred option over intravitreal bevacizumab due to its shorter half-life, resulting in fewer systemic side effects [9].

The prevalence of ROP in Southeast Asia ranges from 11.9% to 40.7% [10]. In Malaysia, the prevalence is 17.4%, making it one of the leading causes of avoidable childhood blindness in the country [11]. There is a lack of research from Southeast Asian countries, like Malaysia, comparing the therapeutic efficacy and outcomes of LPC and IVR for treating type I ROP. To address this, we conducted a study evaluating the treatment efficacy, anatomical outcomes, and refractive results of LPC and IVR in treating type I ROP in Malaysia with a one-year follow-up.

## Materials and methods

This retrospective study evaluates the efficacy of LPC and IVR in treating type I ROP and aggressive ROP (A-ROP) at Hospital Universiti Sains Malaysia, Hospital Selayang, and Hospital Kuala Lumpur in Malaysia. Data collection was carried out between January 2019 and December 2021. The study protocol adhered to the tenets of the Declaration of Helsinki, and ethical approval was obtained from the National Medical Research Register (NMRR) (approval number: NMRR ID-23-01753-OWD (IIR)).

The diagnostic and therapeutic approaches were based on ETROP and International Classification of Retinopathy of Prematurity, Third Edition (ICROP3) study criteria [4,12]. Type I ROP was characterized by the presence of any of the following conditions: ROP in zone I stage 3; zone I stage 1+, 2+, or 3+; and zone II stage 2+ or 3+ [4]. A-ROP is characterized by the accelerated onset of pathological neovascularization and the presence of severe plus disease, without the progression through the conventional stages of ROP [12]. The inclusion criteria include all infants who received either LPC or IVR as the primary treatment for bilateral type I ROP or A-ROP. Children with stage 4 or 5 ROP during the initial diagnosis were excluded from the study.

Type I ROP was a clear indication for treatment, with either LPC or IVR. Zone II ROP, excluding the posterior zone II, was treated with LPC. Meanwhile, IVR was the preferred treatment modality for zone I ROP, posterior zone II, and A-ROP. Treatment with either LPC or IVR was administered within 48 hours of diagnosis in type I ROP and immediately in A-ROP [4]. All treatment effects and systemic concerns were explained based on the existing literature. Informed consent was obtained from all parents before administering LPC or IVR treatment. Both procedures were performed under general anesthesia in the neonatal intensive care unit by the same ophthalmologist in each respective center. LPC involves using an 810 nm diode laser device (Iris Medical OcuLight SLx, IRIDEX Corporation, Mountain View, California, United States) to thoroughly treat the avascular retina from the ridge to the ora serrata in a near-confluent laser burn. Topical antibiotics were given for one week after the LPC. 

IVR (Lucentis, Novartis, Basel, Switzerland) is administered after disinfecting the injection site with 5% povidone-iodine. The eye was stabilized with toothed forceps, and a 30-gauge needle was used to inject 0.25 mg/0.025 mL ranibizumab directly into the vitreous cavity, aiming towards the optic nerve, approximately 1 mm behind the limbus. The intraocular pressure and central artery patency were immediately checked following the injections. Topical antibiotics were given for one week after the IVR. Patients were re-examined the day after either LPC or IVR treatment. Continued examinations were performed weekly for the first month, then two-weekly for three months, and monthly until vascularization of the peripheral retina was achieved in zone III without any active component, including tractional tissues, retinal detachment, or hemorrhage. The examination frequency would revert to weekly if reactivation was detected. 

The efficacy of treatment was determined by the time taken to complete regression, regression rate, and reactivation rate. Regression was defined as the disappearance of ridges, neovascularization, and plus disease, while reactivation was characterized by the reappearance of neovascularization or plus disease after a period of regression. Patients requiring additional LPC within two weeks of the initial treatment due to incomplete laser coverage were not classified as reactivation. 

The anatomical outcome was measured from the time initial treatment was given up until one year of adjusted age to look for retinal detachment or dragging of the optic disc. The refractive outcomes were measured from best-corrected visual acuity (BCVA), spherical equivalent (SE), and myopia at one year of adjusted age. Myopia was defined as a condition where the SE was equal to or more than -0.25 diopters (D). The Cardiff acuity test was employed to measure the BCVA, expressed as the logarithm of minimal angle resolution (logMAR). Cycloplegic refraction was conducted using manifest refraction, and the SE was duly recorded. 

All analyses were performed using a statistical software package (IBM SPSS Statistics for Windows, Version 27.0 (Released 2020; IBM Corp., Armonk, New York, United States)). The Mann-Whitney U-test was utilized to compare continuous variables between the two groups. For comparing categorical variables between groups, either Pearson's chi-squared or Fisher's exact test was employed. A p-value of less than 0.05 was considered statistically significant.

## Results

Ninety-two eyes of 46 infants were included in the study. Among them, 50 eyes of 25 infants received LPC, while 42 eyes of 21 infants were treated with IVR as the primary intervention. Table 1 shows that a higher percentage of infants with cardiovascular disease received IVR treatment (66.7%) compared to LPC treatment (40%) (p<0.05). However, no significant differences were observed in terms of gestational age, birth weight, respiratory distress syndrome, sepsis, and intraventricular hemorrhage (p>0.05) between the two treatment groups.

**Table 1:** Clinical data between groups treated with LPC and IVR The data have been represented as median (IQR) (*), Mann-Whitney U-test (**), Pearson's chi-squared test (‡), Fisher's exact test (†), number (n), and percentage (%) LPC: laser photocoagulation; IVR: intravitreal ranibizumab

	LPC, n=50 (%)	IVR, n=42 (%)	P-value
Gestational age (weeks)	27 (4)*	28 (2)*	0.831**
Birth weight (gram)	1000 (543)*	850 (335)*	0.225**
Respiratory distress syndrome	46 (92.0)	34 (81.0)	0.390†
Cardiovascular disease	20 (40.0)	28 (66.7)	0.045‡
Sepsis	30 (60.0)	34 (81.0)	0.124‡
Intraventricular hemorrhage	14 (28.0)	12 (28.6)	0.966‡

Table 2 details the ROP status at the time of treatment. A higher percentage of patients with type I ROP received LPC compared to IVR (96% vs. 85.7%). Conversely, a greater percentage of patients with A-ROP were treated with IVR than with LPC (14.3% vs. 4%). Additionally, the IVR group had more cases involving zone I. However, there were no significant differences between the LPC and IVR groups regarding the zone and stage of ROP, the presence of plus disease, and the type of ROP.

**Table 2:** The status of ROP at the time of treatment The data have been represented as Pearson's chi-squared test (‡), Fisher's exact test (†), number (n), and percentage (%) ROP: retinopathy of prematurity; LPC: laser photocoagulation; IVR: intravitreal ranibizumab; A-ROP: aggressive retinopathy of prematurity

		LPC, n=50 (%)	IVR, n=42 (%)	P-value
Zone	I	12 (24.0)	18 (42.9)	0.174‡
	II	38 (76.0)	24 (57.1)	
Stage	1	0 (0.0)	4 (9.5)	0.148†
	2	6 (12.0)	10 (23.8)	
	3	44 (88.0)	28 (66.7)	
Plus		38 (76.0)	28 (66.7)	0.484‡
Type I ROP		50 (100.0)	36 (85.7)	0.088†
A-ROP		0 (0.0)	6 (14.3)	0.088†

Table 3 demonstrates that infants treated with LPC had a significantly higher regression rate compared to those treated with IVR (100% vs. 81%, p<0.05). Conversely, the reactivation rate was significantly higher in the IVR group compared to the LPC group (19% vs. 0%, p<0.05). Infants in the IVR group were significantly younger at the time of initial treatment compared to those in the LPC group (13 weeks vs. 19 weeks, p<0.05). At one year of adjusted age, infants treated with LPC had low myopia compared to IVR (SE -0.25 vs. +0.50, p<0.05), and infants treated with IVR had significantly better BCVA compared to LPC (logMAR 0.4 vs. 0.5, p<0.05). LPC-treated infants showed a faster regression rate but exhibited more occurrences of retinal detachment; however, both variables showed no statistical significance. 

**Table 3:** Clinical and refractive outcomes at one year post LPC and IVR The data have been represented as median (IQR) (*), Mann-Whitney U-test, Fisher's exact test (†), number (n), and percentage (%) BCVA: best-corrected visual acuity; SE: spherical equivalent; LPC: laser photocoagulation; IVR: intravitreal ranibizumab; PMA: postmenstrual age

Outcome parameters	LPC, n=50 (%)	IVR, n=42 (%)	P-value
Time to complete regression (week)	7.0 (7.0)*	8.0 (4.0)*	0.418**
Reactivation rate, n (%)	0 (0.0)	8.0 (19.0)	0.037†
Regression rate, n (%)	50.0 (100.0)	34.0 (81.0)	0.037†
PMA of initial treatment (week)	19.0 (29.0)*	13.0 (6.0)*	0.019**
Retinal detachment, n (%)	4.0 (8.0)	0.0 (0.0)	0.493†
Spherical equivalent (diopter)	-0.25 (1.38)*	0.50 (1.38)*	0.022**
BCVA (logMAR)	0.5 (0.1)*	0.4 (0.1)*	0.001**

Table 4 presents the analysis of risk factors associated with type I ROP reactivation after initial treatment. There was no significant association between gestational age, birth weight, plus disease, zone I ROP, and the type of initial treatment received with the reactivation of ROP (p>0.05).

**Table 4:** Risk factors associated with ROP reactivation The data have been represented as odds ratio (OR) and 95% confidence interval (95% CI) LPC: laser photocoagulation; IVR: intravitreal injection of ranibizumab

Variables	Simple logistic regression	
	Crude OR (95% CI)	P-value
Gestational age		
26 weeks or less	1	
More than 26 weeks	1.21 (0.16, 9.42)	0.855
Birth weight		
Less than 1000 g	1	
More than 1000 g	0.68 (0.09, 5.31)	0.713
Plus disease		
Yes	1	
No	3.67 (0.45, 29.76)	0.224
Zone I ROP		
Yes	1	
No	0.45 (0.06, 3.54)	0.447
First treatment choice		
LPC	1	
IVR	0	0.998

## Discussion

This research presents findings on the treatment of type I ROP using either LPC or IVR. The data is sourced from three tertiary centers in Peninsular Malaysia. The study evaluates the treatments' efficacy, anatomical outcomes, and refractive results. Table 5 compares the outcomes of studies conducted in South Korea, the United States, Turkey, Japan, China, and Saudi Arabia and our current study in Malaysia [7,8,13-17]. Generally, ROP infants in the United States have lower median birth weight (MBW) and younger median gestational age (MGA) compared to those in Asian countries. Studies from South Korea, Japan, Turkey, and Saudi Arabia reported a higher prevalence of myopia in eyes treated with LPC compared to those treated with IVR [7,13-15]. Additionally, a study from South Korea found a higher incidence of retinal detachment in infants treated with LPC, which aligns with the findings of our study [7].

**Table 5:** Summary of published studies on ROP treated with LPC or IVR The data have been represented as number (n) and percentage (%) MBW: mean or median birth weight; MGA: mean or median gestational age; RD: retinal detachment; LPC: laser photocoagulation; IVR: intravitreal ranibizumab; IVB: intravitreal bevacizumab; TM: total myopia; SE: spherical equivalent; NR: not relevant

Author (country, year)	Treatment	MBW (gram)	MGA (weeks)	Eyes (n)	TM	SE	Anatomical outcomes (n, %)
Kang et al. [7] (South Korea, 2019)	LPC	1012.0±301.1	28.8±10.3	314	NR	-1.09±3.68	RD: 8 (5%)
	IVR	1049.2±411.1	27.3±2.5			+0.11±3.58	RD: 1 (0.7%)
Stahl et al. [8] (Multicenter, 2019)	LPC	791 (224)	26 (23)	225	NR	NR	NR
	IVR	886 (299)	25 (23)				
Murakami et al. [13] (Japan, 2021)	LPC	722±147	25.7±1.4	52	NR	-0.87±3.14	Strabismus: 21.4%
	IVB	816±369	26.8±2.9			-0.04±0.31	Strabismus: 0%
Gunay et al. [14] (Turkey, 2017)	LPC	1119.47±336.96	28.23±2.50	264	59.6%	-0.81	NR
	IVR	1195.90±466.98	27.95±2.90		31.8%	0.78	
Elabbasy et al. [15] (Saudi Arabia, 2022)	LPC	786.67±164.67	26.26±2.3	69	3 (11.1%)	NR	Strabismus: 8 (29.6%)
	IVR	785.46±175.81	25.48±2.3		3 (7.1%)		Strabismus: 2 (4.8%)
Zhang et al. [16] (China, 2017)	LPC	1060±0.24	28.27±1.84	100	NR	NR	NR
	IVR	1220±0.32	28.96±1.59				
Barry et al. [17] (United States, 2021)	LPC	708.8 (186.8)	24.9 (1.5)	1167	NR	NR	RD: 29 (7.9%)
	IVR	657.9 (170.8)	24.6 (1.7)				RD: 0 (0%)
Current study (Malaysia, 2024)	LPC	1000 (543)	27 (4)	92	26 (52%)	-0.25 (1.38)	RD: 2 (8%)
	IVR	850 (335)	28 (2)		12 (28.6%)	+0.5 (1.38)	RD: 0 (0%)

Recent studies have shown variable results regarding the effectiveness of anti-VEGF treatment for ROP. Some research has postulated that anti-VEGF agents have advantages over LPC, including easier administration, rapid action, and superior refractive and anatomical outcomes [6,8,13,14,18,19]. However, other studies have reported contrasting findings [18,20-22]. Our study revealed a significantly higher rate of regression in the LPC-treated group compared to the IVR-treated group (100% vs. 81%, p<0.05); this contradicts the previous findings reported by Linghu et al. [23]. Conversely, we found a higher reactivation rate in the IVR group compared to the LPC group. This result is consistent with the findings of a meta-analysis conducted by Li et al. [24]. Numerous studies have consistently shown a similar trend over the past decades, postulating a higher incidence of reactivation with intravitreal anti-VEGF treatment compared to LPC [14-16,24]. Xiang et al. proposed a compensatory mechanism theory in ROP, suggesting that when VEGF levels are low, other vascular growth factors are upregulated [25]. Our study indicates that IVR was used to treat a higher number of A-ROP and zone I ROP cases at a younger postmenstrual age, suggesting that the IVR-treated infants had more severe ROP. Such observations could partially explain the higher ROP reactivation incidence observed with IVR treatment.

A higher incidence of retinal detachment was detected following LPC treatment in comparison to IVR (p>0.05). Barry et al. reported that ROP treatment with anti-VEGF agents led to superior short-term anatomical outcomes [17,22]. LPC demonstrated a slightly delayed VEGF-lowering effect as laser ablation of the avascular retina requires time to diminish the production of new VEGF. Conversely, administration of intravitreal anti-VEGF agents directly reduces VEGF levels in the ocular environment, resulting in a more rapid mechanism of action than LPC [26]. In our study, three infants developed retinal detachment between 12 and 18 weeks following their initial IVR treatment. Of these, two infants had unilateral retinal detachment, while one had bilateral retinal detachment. Initially, two of these infants were diagnosed with A-ROP, and one had type I ROP. It was noted that each case involved a documented history of non-compliance with follow-up appointments. This underscores the pivotal role of parental counseling in optimizing the outcomes of ROP treatment.

Our study shows a significant difference in BCVA and SE at one year of age between the LPC and IVR groups. Infants treated with IVR had significantly better median BCVA (logMAR) compared to LPC: 0.4 vs. 0.5 (p<0.05). Conversely, Murakami et al. reported similar BCVA in both treatment groups [13]. Our study observed low myopia in the LPC group and low hyperopia in the IVR group, with values of -0.25 D versus +0.50 D, respectively (p<0.05). In the LPC group, the myopia rate was 52%, whereas in the IVR group, it was 28.6% (p>0.05). Studies conducted in South Korea, Turkey, and Saudi Arabia have shown comparable results, indicating a higher occurrence of myopia among ROP patients treated with LPC [7,14,15]. The exact mechanism of myopia development associated with LPC in ROP patients remains unclear. It has been suggested that the preserved peripheral retina in anti-VEGF-treated eyes may contribute to a normal emmetropization process [27].

The MBW was lower in the IVR group (p>0.05). However, the MGA was nearly equivalent in both groups (p>0.05). Both parameters did not show any statistical significance. These findings are in line with reported data from other studies, where infants with ROP from Asian countries exhibited higher birth weights and older gestational ages compared to those from Western countries [5,14,16-18,28]. The observed differences may be attributed to the improved survival rates of lower birth weight and younger infants in Western or more developed countries. In our study, there was no significant association between lower MBW and ROP reactivation, which contradicts the findings of Zou et al. and Mintz-Hittner et al. [28,29]. Regular follow-ups are crucial to address the late reactivation of ROP.

This study offers valuable insights due to the current lack of comparative data on the outcomes of LPC and IVR for treating type I ROP in Malaysia. However, it is subject to several limitations. These include its retrospective design, which introduced selection bias, a small sample size, inter-observer bias, and a relatively short duration of follow-up. Nonetheless, our study holds significant relevance as there is limited data on the anatomical and refractive outcomes of LPC-treated versus IVR-treated type I ROP in Malaysia. We encourage further research on this less-explored topic within the Malaysian population to enhance treatment strategies for sight-threatening ROP.

## Conclusions

LPC and IVR are both an effective treatment option for infants with type I ROP. While IVR showed better anatomical and refractive outcomes, LPC demonstrated advantages in terms of a lower reactivation rate and faster regression. Infants treated with LPC were found to be significantly more myopic. These results may provide valuable insights into the management of type I ROP and highlight the importance of considering individual patient characteristics when selecting treatment modalities. Further research is necessary to gain a clearer understanding of the long-term outcomes and to optimize treatment strategies for sight-threatening ROP in premature infants.
